# Prognostic Factors Influencing Survival in Ovarian Cancer Patients: A 10-Year Retrospective Study

**DOI:** 10.3390/cancers15245710

**Published:** 2023-12-05

**Authors:** Maria Andreou, Maria Kyprianidou, Christos Cortas, Irene Polycarpou, Demetris Papamichael, Panteleimon Kountourakis, Konstantinos Giannakou

**Affiliations:** 1 Department of Health Sciences, School of Sciences, European University Cyprus, Nicosia 1516, Cyprus; ma212322@students.euc.ac.cy (M.A.); m.kyprianidou@external.euc.ac.cy (M.K.); i.polycarpou@euc.ac.cy (I.P.); 2 Department of Medical Oncology, Bank of Cyprus Oncology Centre, Nicosia 2006, Cyprus; christos.cortas@bococ.org.cy (C.C.); demetris.papamichael@bococ.org.cy (D.P.); p.kountourakis@medihospital.com.cy (P.K.); 3 Department of Medical Oncology, Mediterranean Hospital of Cyprus, Limassol 3117, Cyprus

**Keywords:** ovarian carcinoma, epithelial ovarian cancer, survival, prognostic factors, Cyprus

## Abstract

**Simple Summary:**

The prognosis of ovarian cancer patients is intricately shaped by a multitude of factors, and there exist several pivotal favorable prognostic factors that exert a discernible influence on survival. In this study, we analyzed factors influencing the survival of ovarian cancer patients in Cyprus. We examined data from 106 women with ovarian cancer and found that the FIGO stage, type of surgery, and performance status were significant factors associated with overall survival (OS). Progression-free survival (PFS) was influenced by FIGO stage and performance status. For patients with high-grade serous carcinoma, the performance status, FIGO stage, and type of surgery were initially considered prognostic factors, but in multivariate analysis, only performance status and FIGO stage remained statistically significant for OS. These findings contribute to our understanding of disease and could help improve treatment and patient outcomes.

**Abstract:**

Objective: To analyze the factors associated with overall survival (OS) and progression-free survival (PFS) in patients with ovarian cancer in Cyprus. Methods: We retrospectively analyzed data from patients with histologically confirmed epithelial ovarian cancer (EOC) and primary peritoneal cancer (PPC). Results: A total of 106 women diagnosed with ovarian cancer were included, with a median age at diagnosis of 58 years. The Kaplan–Meier survival analysis showed a median OS of 41 months (95% C.I = 36.9, 45.1), and the FIGO stage (*p* < 0.001), type of surgery (*p* < 0.001) and performance status (*p* < 0.001) were identified as statistically significant prognostic factors for OS. PFS analysis revealed the FIGO stage (*p* = 0.006) and the performance status (*p* < 0.001) as significant prognostic factors. Additionally, a Cox regression analysis for median OS was performed for patients with high-grade serous carcinoma, identifying the performance status, FIGO stage, and type of surgery as prognostic factors in univariate analysis. However, in the subsequent multivariate analysis, the performance status and the FIGO stage were confirmed to be the only statistically significant prognostic factors for OS (*p* < 0.05). Conclusions: This study confirms that the FIGO stage, performance status, and surgery type were considered as prognostic factors for OS in ovarian cancer.

## 1. Introduction

In 2020, ovarian cancer represented 3.4% of new cancer cases and 4.7% of cancer-related deaths in women worldwide. By 2040, there will be a 37% increase in global incidence, with 428,966 new cases expected, accompanied by a significant rise in deaths [1]. It is the seventh most common cancer among women and the eighth most lethal cancer worldwide, making it the most fatal gynecological cancer [2]. Epithelial ovarian cancer (EOC) and primary peritoneal cancer (PPC) are two disease entities that are addressed and treated in an identical approach in clinical practice, since they present a similar histology, natural history, prognosis, and response to medical treatment [3,4].

The etiology of EOC is not fully understood although there are several important risk factors including genetic, chemical, physical, hormonal, and nutritional factors [5,6,7]. There is evidence that the risk of developing ovarian cancer increases with the number of ovulatory cycles a woman has [8,9]. Factors associated with ovulation such as estrogens, progesterone, and gonadotropin-releasing hormones are associated with ovarian cancer. High levels of gonadotropin-releasing hormones during ovulation may promote ovarian epithelial cell proliferation and tumor growth. In addition, there is a link between ovarian cancer and estrogen levels, which change during ovulation [8,9]. Consequently, early onset of menstruation, delayed menopause, infertility, high levels of gonadotrophins, and postmenopausal hormone intake can affect ovulation and may increase the risk of developing the disease [5,6]. Women who carry an inherited deleterious mutation in the BRCA1 or BRCA2 gene face the highest risk of developing ovarian cancer [10]. Conversely, factors like parity, age at first and last pregnancy in multiparous women, multiple pregnancies, breastfeeding, hormonal contraception, and hysterectomy seem to have a protective effect [5,11,12,13,14].

The prognosis for patients with ovarian cancer is influenced by various factors. Several important favorable prognostic factors that impact overall survival include good performance status, parity histological type other than mucinous or clear cells, well-differentiated tumor, early-stage disease, absence of ascites, smaller disease volume prior to surgical removal, and smaller residual tumor volume after primary surgery [15,16,17,18]. It is important to note that while the mucinous and clear cell histological types are generally associated with favorable outcomes, this is primarily driven by frequent early-stage diagnoses in these histotypes [19,20,21,22]. However, in advanced-stage cases, mucinous and clear cell ovarian cancer may have markedly poor outcomes [23]. Genetic predisposition is a significant prognostic factor for the onset of the disease [24]. The diagnosis of ovarian cancer at an early stage is challenging due to the fact that is mostly asymptomatic or it presents with non-specific symptomatology. Hence, ovarian cancer is mostly diagnosed in the advanced stage, and it remains an unresolved global health problem [25,26].

The peritoneum, which is the thin layer of tissue lining the abdomen, is developed by epithelial cells, the same cells as on the surface of the ovary. Consequently, PPC and EOC, the most common type of ovarian cancer, exhibit striking similarities [3,4]. PPC is a relatively uncommon disease that originates in the peritoneum itself [27]. EOC and PPC are histopathologically classified to grade serous, endometrioid, mucinous, and clear cell carcinomas. The most common subtype is the serous type, which is further classified into high-grade serous carcinoma and low-grade serous carcinoma. The remaining subtypes are graded as Grade I, II, and III according to tumor cell differentiation [28].

The workup and treatment of ovarian cancer are based on a multimodal approach, primarily involving surgery and systemic treatment with chemotherapy, monoclonal antibodies, and targeted therapies. The treatment approaches for ovarian cancer depend on the histologic type, grade, and stage of the disease, typically encompassing a combination of surgery and chemotherapy [29]. The treatment of EOC and PPC is similarly based on a multimodality approach. Surgical intervention for the removal of the primary site and debulking surgery in advanced disease is the backbone of the treatment. Systemic therapy in an adjuvant or neoadjuvant setting is administrated in most of the cases with exceptions in some cases of early disease [29,30,31]. Thus, the main treatment objective is achieving optimal debulking through surgical intervention. This can be accomplished ideally with primary surgical debulking (PDS) or, alternatively, after the patient receives induction chemotherapy to reduce the disease volume, increasing the chances of optimal debulking surgery (IDS). Following the IDS operation, the patient continues with further systemic treatment. The decision regarding IDS or PDS is based on surgical criteria, radiological criteria, and the patient’s performance status [22]. Patients who are medically unfit for surgery receive palliative systemic treatment or are set in best supportive care. The standard chemotherapy regimen, whether adjuvant or neoadjuvant, typically consists of a combination of carboplatin and paclitaxel, with the addition of bevacizumab, an anti–vascular endothelial growth factor (VEGF) antibody, or poly (ADP-ribose) polymerase (PARP) inhibitors in stage III or IV disease [29,31].

The clinicopathological and prognostic characteristics of patients with ovarian cancer in Cyprus are currently not well understood. While there are a few studies in the literature that explore hereditary ovarian cancer and gene mutations of BRCA1 and BRCA2 [32,33,34], research specifically focusing on the prognostic factors of ovarian cancer has not been conducted in Cyprus. Thus, the aim of this study was to investigate and examine the factors that are linked to the overall survival (OS) and progression-free survival (PFS) of individuals diagnosed with ovarian cancer in the republic of Cyprus. Such information is crucial for understanding the disease profile in a specific population, stratifying patients, and developing personalized treatment strategies to improve patient survival rates.

## 2. Materials and Methods

### 2.1. Study Design and Setting

We conducted a retrospective study involving patients with EOC and PPC who received treatment at the Bank of Cyprus Oncology Center (BOCOC) in Nicosia, Cyprus. The BOCOC served as the primary oncology center in the country during that time, catering to approximately 70% of cancer patients in the republic of Cyprus. The present study followed the Equator STROBE (Strengthening the Reporting of Observational studies in Epidemiology) guidelines [35].

### 2.2. Patient Population

This study included data from adult (age ≥ 18 years) patients with histologically confirmed ovarian cancer, without restrictions based on other demographic or clinical characteristics. Recurrent EOC and PPC, as well as advanced and/or metastatic EOC and PPC, were included. Patients with metastatic tumors in the ovaries originating from another primary tumor, incomplete medical records, unconfirmed diagnoses, and those with borderline tumors in the ovaries were excluded.

### 2.3. Data Collection and Variables

The medical records of patients who met the inclusion criteria were thoroughly reviewed. To protect the privacy of the participants, a sequential identification number was employed within the database rather than utilizing names or any other personally identifiable details. Data were extracted for patients diagnosed with ovarian cancer at the BOCOC between 14 October 2010 and 8 August 2019. The patients were followed-up until 31 October 2022. Clinicopathological data, which included age at diagnosis, FIGO stage, cancer type, grade, type of surgery (PDS, IDS, or no surgery), performance status according to the Eastern Cooperative Oncology Group (ECOG), and OS in months, were collected. For grade classification, patients with ovarian serous carcinoma were categorized as low or high grade, while patients with other cancer types (e.g., mucinous, endometrioid, clear cell, transitional cell, carcinosarcoma, mixed, undifferentiated) were classified as Grade I, II, or III.

### 2.4. Ethical Approval

This study was conducted in accordance with the Helsinki Declaration and was approved by the Cyprus National Bioethics Committee with a waiver of informed consent due to the retrospective, observational design of this study (ΕΕΒΚ ΕΠ 2020.01.43). Patient anonymity and identity protection were ensured during data collection.

### 2.5. Statistical Analysis

The distribution of continuous variables was assessed using the Shapiro–Wilk normality test. Participant characteristics are presented as mean ± standard deviation (SD) for normally distributed continuous measures, and as median (1st quartile, 3rd quartile) for continuous measures that do not follow a normal distribution. Categorical variables are reported as absolute counts (n) and relative frequencies (%). The chi-square test of independence was employed to examine associations between categorical variables. For comparisons involving more than two categories, the Kruskal–Wallis’s rank test was used to evaluate the average level of continuous baseline characteristics without a normal distribution. Survival was defined as the time from diagnosis until either death or until the time of the last follow-up. Kaplan–Meier curves were used to demonstrate the OS and PFS, and the *p*-values of the log-rank test were used to assess significance. The OS was calculated from the time of cancer diagnosis, wherein the day of diagnosis was defined as the date that the histologic specimen (tissue biopsy or surgical specimen) confirmed a cancer of epithelial origin. Cases were censored at their last follow-up. The Kaplan–Meier curves were stratified according to age group, FIGO stage and performance status. Univariate and multivariate Cox proportional hazards models were utilized to identify factors of prognostic significance. Interactions between covariates and time-varying effects were tested, and none were found. All statistical tests were two-sided, and a significance level of α = 0.05 was set. The statistical analysis was conducted using STATA 14.0 and IBM SPSS 24.0 software.

## 3. Results

### 3.1. Population Description

A total of 106 women were included in this study. The median age at the time of diagnosis was 58 years old (IQR 49–64 years); most participants were less than 60 years old (*n* = 62, 58.5%). Most patients were diagnosed with FIGO Stage III (*n* = 63, 59.4%), followed by Stage IV (*n* = 23, 21.7%), Stage I (*n* = 12, 11.3%), and Stage II (*n* = 8, 7.6%). In patients with ovarian serous carcinoma, the majority had a high-grade status (*n* = 75, 73.5%), while the remaining had non-serous ovarian cancer (*n* = 27, 26.5%). Specifically, among those who had non-serous ovarian cancer, four (14.8%) had low-grade serous, eight (29.7%) had mucinous, five (18.5%) had endometrioid, one (3.7%) had clear cell, six (22.2%) had mixed, and three (11.1%) had undifferentiated cancers. Among patients with non-serous carcinomas, approximately 50% were at grade II (*n* = 12, 48.0%), and more than half were at grade III (*n* = 13, 52.0%). Furthermore, 69.8% of the patients underwent PDS, 24.5% underwent IDS, and only 5.7% did not undergo surgery. Most patients had a performance status greater than 0 (*n* = 64, 61.5%) (Table 1). Supplementary Table S1 provides more detailed information on the clinical and histopathological characteristics of low-grade serous and non-serous ovarian cancer patients.

### 3.2. Overall Survival

**Figure 1:**
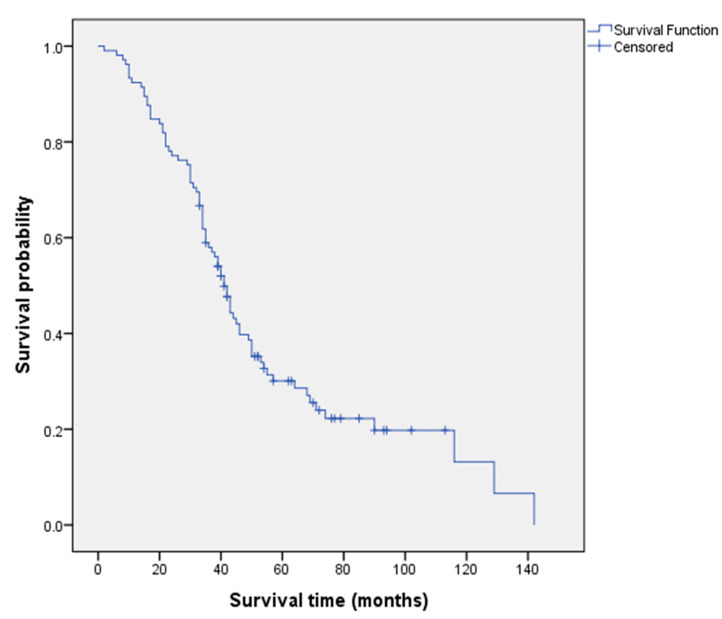

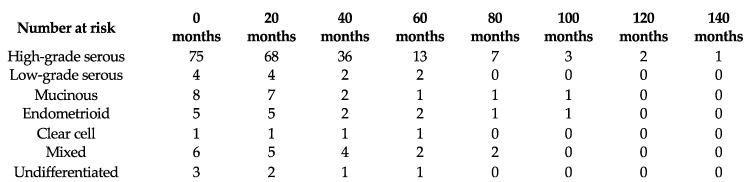
Kaplan–Meier overall survival analysis for all patients.

Figure 1 presents the Kaplan–Meier survival analysis for all patients, with a median survival of 41 months (95% confidence interval = 36.9, 45.1).

**Figure 2:**
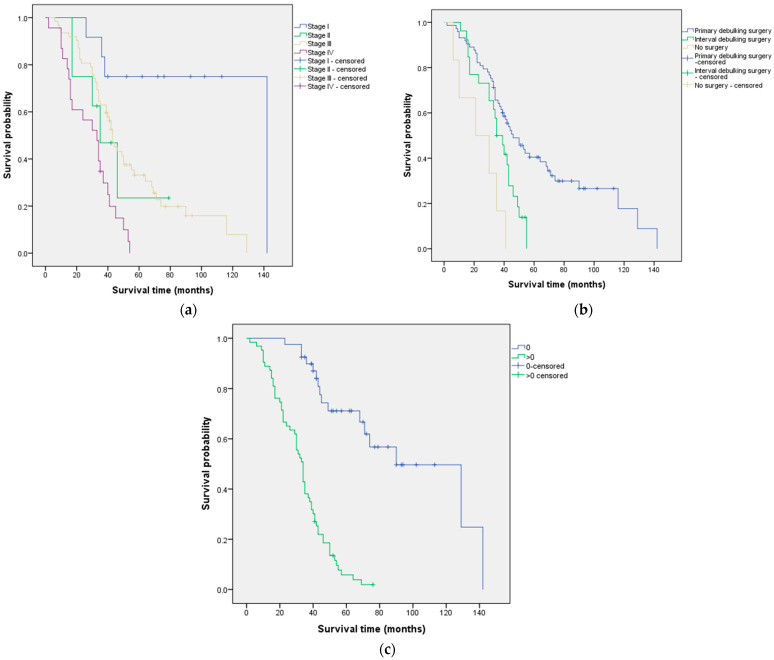
Median overall survival according to (**a**) FIGO stage, (**b**) type of surgery, and (**c**) performance status.

Bivariate analysis (Table 1) identified three prognostic factors associated with OS: FIGO stage (*p* < 0.001), type of surgery (*p* < 0.001), and performance status (*p* < 0.001). The median OS according to these prognostic factors is shown in Figure 2.

Cox regression analysis for median OS was performed for patients with high-grade serous ovarian carcinoma. Univariate analysis identified the performance status, FIGO stage, and type of surgery as prognostic factors for OS. The performance status and FIGO stage remained statistically significant prognostic factors in the multivariate analysis for OS (*p*-values < 0.05) (Table 2). The interactions between the covariates and time-varying effects were not statistically significant, so a stratified analysis was not conducted.

### 3.3. Progression-Free Survival

**Figure 3:**
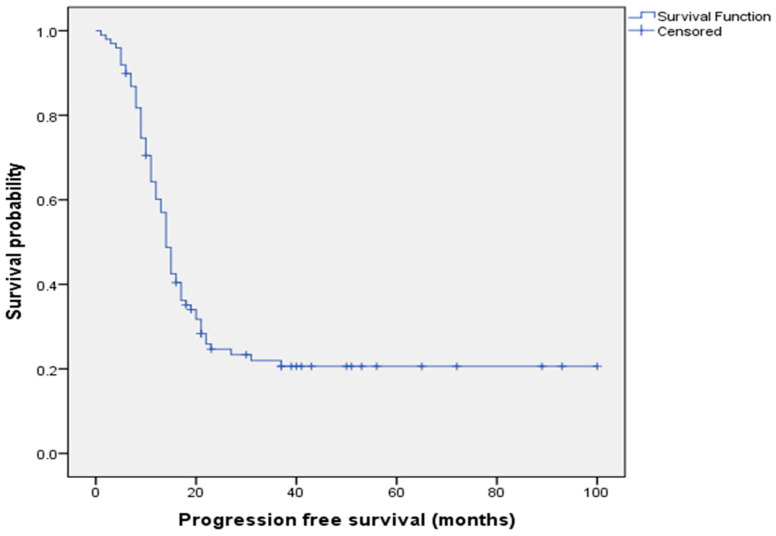
Kaplan–Meier progression-free survival analysis for all patients.

Figure 3 displays the Kaplan–Meier curve of PFS analysis for all patients.

**Figure 4:**
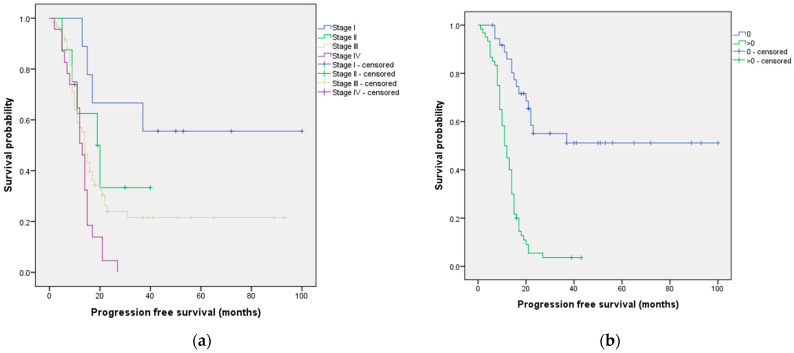
Median progression-free survival according to (**a**) FIGO stage and (**b**) performance status.

Two prognostic factors associated with PFS were identified: FIGO stage (*p* = 0.006) and performance status (*p* < 0.001) (Table 1). The median PFS according to these significant prognostic factors is presented in Figure 4.

## 4. Discussion

In this retrospective study conducted at a single center in Cyprus, we aimed to investigate the factors associated with OS and PFS in patients diagnosed with ovarian cancer. Our study revealed that the performance status, type of surgery, and FIGO stage were independent prognostic factors for OS in patients with high-grade serous ovarian carcinoma. Furthermore, when considering all predictors simultaneously, the performance status and the FIGO stage remained significant prognostic factors for OS.

The Kaplan–Meier survival analysis showed a median OS of 41 months for ovarian cancer patients in our study. It is important to note that some previous studies have reported higher median OS rates. For instance, a retrospective study conducted by the Hellenic Cooperative Oncology Group from February 1976 to December 2006, which included 1791 patients, reported a median OS of 51 months [36]. Another retrospective study focusing on 276 patients with cancer of the corpus uteri reported a median OS of 46 months [37]. The disparity in median OS between our study and these previous studies could be attributed to differences in the sample sizes, populations, and types of ovarian cancer included in those studies. In our study, the median age of diagnosis was 58 years old, which is similar to other studies reporting median ages of 64 years old [38], 61 years old [37], 60 years old [36,39], 58.5 years old [40], and 52.8 years old [16]. However, a study that included 221 patients with stage I or stage II ovarian cancer reported a lower median age at diagnosis of 47 years old [41]. In addition to age at diagnosis, many studies have also reported a significant association between patient age and OS [36,37,38,39,40,42].

Furthermore, our study results demonstrated that, as the FIGO stage increased, the median OS progressively decreased. This finding is consistent with previous studies that have also identified FIGO stage as a prognostic factor for OS [36,42,43,44]. For example, a study analyzing 41,120 cases of endometrial cancer concluded that the FIGO stage influences the survival probability [45]. Additionally, research has shown that patients with advanced disease (higher FIGO stage) have nearly five times the probability of death compared to patients with FIGO stage I [37]. Moreover, our study revealed that patients with a performance status of 0 had a median OS of 90 months, while those with a performance status greater than 0 had a median OS of 34 months. This finding aligns with other studies that have identified performance status as a prognostic factor for OS [36,46]. The Gynecologic Oncology Group also reported that performance status is an independent predictor of OS based on the analysis of 726 females with Stage III or Stage IV epithelial ovarian cancer treated in two randomized trials [47]. Additionally, various studies have noted that performance status is associated with multiple survival outcomes in ovarian cancer patients [15,16,17,48]. In our specific analysis focusing on patients with high-grade serous ovarian carcinoma, a Cox regression analysis was conducted to evaluate the median OS. The multivariate analysis identified the performance status and the FIGO stage as statistically significant prognostic factors for OS, with *p*-values below 0.05. These findings emphasize the importance of considering the performance status and the FIGO stage when assessing OS in patients with high-grade serous ovarian carcinoma.

We also identified important predictors of PFS in our study. Both the FIGO stage and the performance status showed significant associations with PFS. The median PFS varied among different FIGO stages, with stage I demonstrating the longest median survival of 60 months, while stage IV had the shortest median survival of 13 months. These findings are consistent with three recent studies that investigated prognostic factors for PFS and also identified FIGO stage as a significant factor [37,41,49]. For example, a study analyzing data from 662 patients with cervical cancer at stage IB or stage IIA treated at Zhejiang Cancer Hospital between 2008 and 2011 concluded that the 2018 FIGO staging system is crucial for estimating patients’ prognosis after radical surgery in cervical cancer [49]. Similarly, a retrospective study of 276 patients with cancer of the corpus uteri reported that the 5-year disease-free survival rate for patients with FIGO stage I was 91%, while, for patients with FIGO stage II, III, or IV, it was 52.5% [37]. Additionally, according to our findings, patients with a performance status of 0 had a significantly longer median PFS of 58 months compared to those with a performance status greater than 0, who had a median PFS of 11 months. Similar results were reported in a retrospective review that included 1895 patients with stage III epithelial ovarian cancer who underwent primary surgery [17]. Specifically, they found that the median PFS was shorter for patients with a performance status greater than 0 compared to those with a performance status of 0 [17].

The findings of this study on prognostic factors in ovarian cancer have significant implications for clinical practice, public health, and research. The performance status, FIGO stage, and type of surgery were identified as independent prognostic factors for OS in patients with high-grade serous ovarian carcinoma. Healthcare professionals and researchers can use this information to improve risk stratification, personalize treatment approaches, and optimize patient outcomes. This study reaffirms the importance of the FIGO stage in predicting survival outcomes in ovarian cancer. Higher FIGO stages were associated with decreased OS and PFS. Early detection, accurate staging, and timely intervention are crucial for improving outcomes. These findings provide a basis for further research. Future studies can explore additional prognostic factors, investigate novel therapeutic strategies, and evaluate the impact of multidisciplinary care approaches on patient outcomes. Collaborative efforts between researchers, clinicians, and public health agencies can contribute to advancing the understanding and management of ovarian cancer.

Of course, this study is not without its limitations. This study utilized a retrospective design, which inherently relies on existing data and medical records. Consequently, our study may be subject to limitations such as incomplete or missing information and the inability to establish causality. Also, the lack of quality control during the data collection can be considered as a limitation. This study was conducted at a single center, the BOCOC, which may limit the generalizability of the findings. The patient population and treatment practices at the BOCOC may not fully represent the diversity of ovarian cancer patients and their treatments in other regions or healthcare settings. Nevertheless, the BOCOC played a central role as the primary oncology center in the country during the study period, catering to around 70% of cancer patients in Cyprus. In addition, we excluded certain groups of patients, such as those with incomplete medical records, unconfirmed diagnoses, and borderline tumors in the ovaries. These exclusion criteria may introduce bias and limit the generalizability of the findings to these specific patient groups. Also, we did not include cases of primary fallopian tube cancers due to the historical challenges faced by histopathologists in differentiating these cases from ovarian carcinomas. Although we acknowledge their significance as a distinct subtype of gynecological malignancy, their omission is a limitation of our dataset. Furthermore, we acknowledge that the absence of a contemporary pathology review could influence the accuracy and consistency of the pathological data. In addition, our study did not adjust for variations in first-line treatment modalities, including the use of maintenance therapies such as PARP inhibitors (PARPi) or bevacizumab (Bev), which may impact survival outcomes [50,51,52]. Finally, our study is limited by the lack of data concerning the extent of residual disease after surgery, a potential factor associated with survival time in ovarian cancer [53,54,55]. The absence of these data in our analysis may have impacted our findings, underscoring the importance of their inclusion in future research. We stress the significance of acknowledging these limitations as they can affect the generalizability and comprehensive understanding of our findings. Consider these limitations when interpreting and applying our results to clinical practice or further ovarian cancer research.

## 5. Conclusions

In summary, this retrospective study investigated factors associated with OS and PFS in patients diagnosed with ovarian cancer, specifically focusing on high-grade serous ovarian carcinoma. This study identified several significant prognostic factors, including performance status, type of surgery, and FIGO stage. Overall, this study provides valuable insights into the prognostic factors for OS and PFS. The results contribute to the understanding of this disease and can aid healthcare professionals in optimizing treatment approaches and improving patient outcomes.

## Figures and Tables

**Table 1:** Clinical and histopathological characteristics of patients with epithelial ovarian cancer and primary peritoneal cancer.

Characteristics	Number of Patients (%)	Median Overall Survival (Months) (95% C.I)	*p*-Value ^3^	Progression-Free Survival (Months) (95% C.I)	*p*-Value ^3^
Median age at the time of diagnosis (IQR)	58 (49–64)	-	-	-	-
Age group ^1^					
Less than 60 years old	62 (58.5)	44.0 (38.2, 49.8)	0.399	16.0 (10.8, 21.2)	0.345
More than 60 years old	44 (41.5)	39.0 (31.7, 46.3)		12.0 (9.8, 14.2)	
FIGO stage ^1^					
Stage I	12 (11.3)	142.0 (84.0, 145.6)	**<0.001**	60.0 (38.2, 90.8)	**0.006**
Stage II	8 (7.6)	35.0 (20.0, 50.0)		19.0 (8.3, 29.7)	
Stage III	63 (59.4)	43.0 (38.1, 47.9)		14.0 (11.9, 16.1)	
Stage IV	23 (21.7)	33.0 (21.3, 44.7)		13.0 (11.1, 14.9)	
Histologic type ^2^					
High-grade ovarian serous carcinoma	75 (73.5)	41.0 (36.8, 45.2)	0.338	14.0 (12.6, 15.3)	0.658
Other types	27 (26.5)	42.0 (28.3, 55.7)		17.0 (10.3, 23.7)	
Type of surgery ^1^					
Primary debulking surgery	74 (69.8)	46.0 (34.7, 57.3)	**<0.001**	15.0 (11.8, 18.3)	0.065
Interval debulking surgery	26 (24.5)	35.0 (27.8, 42.2)		14.0 (12.8, 15.2)	
No surgery	6 (5.7)	21.0 (0.0, 45.0)		9.0 (0.0, 18.6)	
Performance status ^2^ (ECOG)					
0	40 (38.5)	90.0 (53.6, 126.4)	**<0.001**	58.0 (45.2, 74.0)	**<0.001**
>0	64 (61.5)	34.0 (30.1, 37.8)		11.0 (9.1, 12.9)	

Abbreviations: ECOG: Eastern Cooperative Oncology Group; 95% C.I: 95% confidence interval; ^1^ *n* = 106; ^2^ *n* = 102; ^3^ Kruskal–Wallis test was performed to identify differences in the median survival time among the categories of the characteristics; bold values indicate statistically significant differences at a statistical significance level of <0.05.

**Table 2:** Cox regression analysis for median overall survival in high-grade serous ovarian carcinoma patients (*n* = 75).

	Univariate		Multivariate	
	HR (95% C.I)	*p*-Value	HR (95% C.I)	*p*-Value
Age at the time of diagnosis	1.02 (1.00, 1.05)	0.062	1.02 (1.00, 1.05)	0.073
Performance status (ECOG)				
0	*Ref*		*Ref*	
>0	0.16 (0.08, 0.34)	**<0.001**	0.19 (0.08, 0.44)	**<0.001**
FIGO stage				
Stage I	*Ref*		*Ref*	
Stage II	0.05 (0.01, 0.36)	**0.003**	0.06 (0.07, 0.49)	**0.009**
Stage III	0.40 (0.12, 1.39)	0.151	0.78 (0.21, 2.95)	0.719
Stage IV	0.49 (0.27, 0.88)	**0.017**	0.54 (0.29, 1.00)	**0.05**
Type of surgery				
No surgery	*Ref*		*Ref*	
Primary debulking surgery	2.25 (0.90, 5.60)	0.082	1.89 (0.72, 4.94)	0.197
Interval debulking surgery	0.50 (0.29, 0.84)	**0.009**	0.80 (0.47, 1.37)	0.418

Abbreviations: C.I: confidence interval; ECOG: Eastern Cooperative Oncology Group; bold values indicate statistically significant differences at a statistical significance level of <0.05.

## Data Availability

The data underlying this article will be shared upon reasonable request to the corresponding author.

## Supplementary Materials

The following supporting information can be downloaded at https://www.mdpi.com/article/10.3390/cancers15245710/s1: Table S1: Clinical and histopathological characteristics of low-grade serous and non-serous ovarian cancer patients.
