# Supplementary material for: Prognostic Factors Influencing Survival in Ovarian Cancer Patients: A 10-Year Retrospective Study
Source: Cancers (Basel). 2023 Dec 5;15(24):5710. doi: 10.3390/cancers15245710 (PMC10742060; doi:10.3390/cancers15245710)
Supplement: Supplementary file 1 [file cancers-15-05710-s001.zip › cancers-2695589-supplementary.pdf]

Table S1. Clinical and histopathological characteristics of low-grade serous and non-serous ovarian cancer patients.

Characteristics	No. of patients n (%)	Histology type					
		Low-grade serous n (%)	Mucinous n (%)	Endometrioid n (%)	Clear cell n (%)	Mixed n (%)	Undifferentiated n (%)
**Age group**							
Less than 60 years old	16 (59.3)	3 (75.0)	3 (37.5)	4 (80.0)	1 (100.0)	3 (50.0)	2 (66.7)
More than 60 years old	11 (40.7)	1 (25.0)	5 (62.5)	1 (20.0)	0 (0.0)	3 (50.0)	1 (33.3)
**FIGO stage**							
Stage I	6 (22.2)	1 (25.0)	3 (37.5)	1 (20.0)	1 (100.0)	0 (0.0)	0 (0.0)
Stage II	2 (7.4)	0 (0.0)	1 (12.5)	1 (20.0)	0 (0.0)	0 (0.0)	0 (0.0)
Stage III	14 (51.9)	2 (50.0)	3 (37.5)	2 (40.0)	0 (0.0)	4 (66.7)	3 (100.0)
Stage IV	5 (18.5)	1 (25.0)	1 (12.5)	1 (20.0)	0 (0.0)	2 (33.3)	0 (0.0)
**Type of surgery**							
Primary debulking surgery	23 (85.2)	1 (100.0)	3 (60.0)	4 (100.0)	4 (66.7)	8 (100.0)	3 (100.0)
Interval debulking surgery	4 (14.8)	0 (0.0)	2 (40.0)	0 (0.0)	2 (33.3)	0 (0.0)	0 (0.0)
**Performance status**							
0	14 (56.0)	1 (100.0)	2 (50.0)	3 (75.0)	4 (66.7)	3 (42.9)	1 (33.3)
>0	11 (44.0)	0 (0.0)	2 (50.0)	1 (25.0)	2 (33.3)	4 (57.1)	2 (66.7)
